# Double blind randomized placebo-controlled trial on the effects of testosterone supplementation in elderly men with moderate to low testosterone levels: design and baseline characteristics [ISRCTN23688581]

**DOI:** 10.1186/1745-6215-7-24

**Published:** 2006-08-03

**Authors:** Hamid Reza Nakhai Pour, Marielle H Emmelot-Vonk, Marja Sukel-Helleman, Harald JJ Verhaar, Diederick E Grobbee, Yvonne T van der Schouw

**Affiliations:** 1 Julius Center for Health Sciences and Primary Care, Utrecht Medical Center, Utrecht, The Netherlands; 2 Department of Geriatrics, University Medical Center, Utrecht, The Netherlands

## Abstract

In ageing men testosterone levels decline, while cognitive function, muscle and bone mass, sexual hair growth, libido and sexual activity decline and the risk of cardiovascular diseases increase. We set up a double-blind, randomized placebo-controlled trial to investigate the effects of testosterone supplementation on functional mobility, quality of life, body composition, cognitive function, vascular function and risk factors, and bone mineral density in older hypogonadal men.

We recruited 237 men with serum testosterone levels below 13.7 nmol/L and ages 60–80 years. They were randomized to either four capsules of 40 mg testosterone undecanoate (TU) or placebo daily for 26 weeks. Primary endpoints are functional mobility and quality of life. Secondary endpoints are body composition, cognitive function, aortic stiffness and cardiovascular risk factors and bone mineral density. Effects on prostate, liver and hematological parameters will be studied with respect to safety.

Measure of effect will be the difference in change from baseline visit to final visit between TU and placebo. We will study whether the effect of TU differs across subgroups of baseline waist girth (< 100 cm vs. ≥ 100 cm; testosterone level (<12 versus ≥ 12 nmol/L), age (< median versus ≥ median), and level of outcome under study (< median versus ≥ median).

At baseline, mean age, BMI and testosterone levels were 67 years, 27 kg/m^2 ^and 10.72 nmol/L, respectively.

## Background

In men after the age of 30–40, testosterone production gradually declines which continuously persists into old age [1,2]. Ageing in men is accompanied by a decrease in muscle and bone mass, cognitive changes and decreased libido and sexual activity, all of which have been suggested to be related to the decrease in testosterone production [3]. Recent research has provided evidence that androgens play distinct roles in aspects of bone metabolism[4], body composition such as muscle and fat mass distribution [5,6], cognitive functioning [7], well-being [8], cardiovascular diseases [9], prostate hyperplasia [10,11]and in aspects of sexual behavior [12]. Since androgens are associated with muscle function and with cognitive functioning, it is reasonable to expect androgens to be related to activities of daily living (ADL) as well [13]. The association of lower testosterone levels with age-related conditions and the steady androgen levels decline with aging stimulated further investigations to test usefulness and safety of androgen supplementation.

There are varying degrees of evidence regarding potential risks and benefits of testosterone treatment in older men [14]. The results of the Women Health Initiative (WHI) randomized trial raised concerns about the risk-benefit ratio of hormonal treatment [15]. Concerns regarding the risks have focused primarily on the potential for increased incidence of prostatic cancer, benign prostatic hyperplasia [16], urinary obstruction [17], gynecomastia [18], sleep apnea [19] and polycythemia [20]. Although androgens are necessary for the development and normal function of the human prostate, the role of testosterone in the progression of prostate cancer and benign prostatic hyperplasia is not yet clear. Epidemiological studies have been unable to relate the occurrence of benign prostatic hyperplasia and prostate cancer to androgens [21,22]. The current opinion is that androgens are not causal but permissive for the development of these diseases. Withdrawal of androgens leads to objective response rates of metastatic lesions and of the primary tumor [23]. Recently, the issue whether physiological levels of androgens are associated with prostate cancer risk seems to be adequately refuted by the quantitative assessment of the current evidence [22]. In spite of the circumstantial scientific suggesting potential risks and limited support for benefits, testosterone use has become increasingly popular in men of all ages.

The levels at which testosterone therapy might be indicated in subjects with particularly low circulating levels are also unclear. It is uncertain whether men who are at the lower end of the normal range of testosterone production would benefit from treatment. Therefore, we set up a randomized trial with testosterone undecanoate. The key objectives of the study are to treat men aged 60 years and over, with low to low-normal testosterone levels, with testosterone undecanoate during six months and to study the effect of this treatment on functional mobility, quality of life, body composition, cognitive function, vascular ageing and bone mineral density. Safety assessments will performed by measurements of the prostate, liver enzymes and hematological parameters.

Testosterone undecanoate, the oral androgen to be used in this intervention study, did not lead to signs of prostate tumors in men who were followed-up for a minimum of 10 years [24]. With the androgens used in this study androgen levels do not rise above normal. Most benefits are expected on muscle strength and cognitive functioning. These areas are of major importance in determining the ability of living independently at old age, and therefore elderly men are a potential subgroup that could benefit from androgen supplementation.

## Design and methods

The study is a single center randomized, placebo-controlled, double-blind trial to assess the effects of supplementation with testosterone undecanoate on functional mobility, quality of life, body composition, cognitive function, vascular function and risk factors, and bone mineral density in men, aged 60–80 years. The planned number of study subjects is 240. After completion of the baseline measurements subjects are randomized to four capsules of 40 mg testosterone undecanoate (TU) or placebo daily for 26 weeks. The Institutional Review Board of the University Medical Center Utrecht approved the study protocol. All participants gave written informed consent at screening visit.

### Study population

The study was designed so that the study population would comprise men in the lower half of the population distribution of testosterone levels. Therefore, the exclusion criteria were mainly limited to criteria indicating contraindications for testosterone, a high probability of experiencing serious side effects, or a low likelihood of completing the study.

### In and exclusion criteria

Participants in this trial were healthy men aged 60 to 80 years (at the moment of inclusion), who lived in Utrecht and vicinity and had a low normal testosterone concentration. Detailed information and definitions of the exclusion criteria are displayed in Table 1. Men could not be enrolled when they had a history of recent severe myocardial infarction or cerebrovascular accident (< 6 months), cardiac failure, unless medically treated and not symptomatic, history or presence of any malignancy within the past 5 years, except for non-melanoma skin cancer, subjects with ever history of testosterone (any hormone) dependent tumors (especially prostate or breast cancer), serious liver disease, serious renal disease, hematological abnormalities, epilepsy (or the use of anti-epileptic medication) or migraine more than once a month, diabetes mellitus, presence of any disease or condition that is clinically relevant and which might result in premature discontinuation, according to the opinion of the investigator, corticosteroid use, use of testosterone esters and alike substances within the past 60 days, increased age-specified-PSA levels and prostate hypertrophy in medical history (table 1).

**Table 1:** Exclusion criteria

*Disease/Condition*	*Definition*
Recent severe myocardial infarction or cerebrovascular accident	< 6 months
	
Cardiac failure, unless medically treated and not symptomatic	
	
History or presence of any malignancy within the past 5 years, except for non-melanoma skin cancer. Subjects with ever history of testosterone (any hormone) dependent tumors.	
	
Serious liver disease	ASAT, ALAT, AF, γGT >3 times upper limit of reference value (ASAT: 15–45 U/L; ALAT: 10–50 U/L; AF: 40–130 U/L ; γGT: 15–70 U/L:
	Central Laboratory, UMC Utrecht)
	
Serious renal disease	Serum creatinine levels > 180 mol/L)
	
Epilepsy (or the use of anti-epileptic medication) or migraine > once a month	
	
Diabetes mellitus	Diagnosed by physician or fasting glucose level of 6.9 mmol/L or higher (capillary)
	
Presence of any disease or condition that is clinically relevant and which might result in premature discontinuation, according to the opinion of the investigator.	

*Medication*	*Definition*

Corticosteroid use	Orally: <6 months ago in dosage >7.5 mg a day, with the exception of short bouts of prednisone for the period of 7 days. Inhalation: <6 months ago in the dosage of >800 g a day)
	Use of testosterone esters and alike substances within the past 60 days
	
Conditions for which increase of androgen-like substances are contra-indicated	PSA levels: age 60–69 years >4.5 μg/l;70 years and over >6.5 μg/L
	Prostate hypertrophy in medical history Renal, liver function abnormalities or hematological abnormalities (Hb <7; Ht >0.50)
	Prostate or breast cancer

### Recruitment

**Figure 1:**
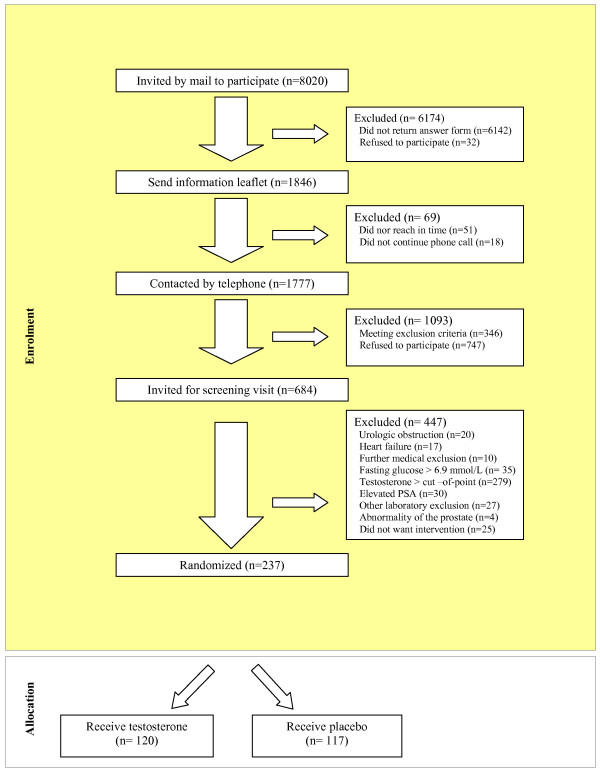
Participants flow diagram.

Recruitment started in November 1st, 2003. Participants were recruited by direct mailing to 8020 men aged 60 to 80 years whose addresses were randomly selected by the municipal register of Utrecht. 1,846 men expressed interest in the study by sending in a response-card. Men who responded were explained the aim of the study and any questions were answered. Also an information leaflet was mailed. In this leaflet explanation was given regarding the study and the in – and exclusion criteria protocol. After one week of evaluation time 1,777 men were contacted by telephone and invited to the information and screening visit. Of these, 1030 responded positively and 747 refused to participate. Also most of the exclusion criteria were checked by telephone. Of those 1030 men, 346 were excluded because of one or multiple exclusion criteria (Fig. 1).

### Information and screening visit

The selected 684 participants were asked to attend the ambulatory clinic of the Julius Center for Health Sciences and Primary Care of the University Medical Center Utrecht, The Netherlands for more information about study and screening. During this visit, after answering remaining questions, the informed consent form was signed. The participants filled in two questionnaires; the Androgen Deficiency in Ageing Males (ADAM) questionnaire and the Ageing Males' Symptoms rating scale (AMS). Remaining possible exclusion criteria were checked through a medical history and blood examination in the following order: fasting glucose level (capillary) of ≥ 6.9 mmol/L, (n = 35 exclusions), testosterone level higher than the 50^th ^percentile of the study population-based testosterone distribution (n = 279 exclusions), elevated PSA level (age 60–69 years ≥ 4.5 μg/L; 70 years and above ≥ 6.5 μg/L (n = 30 exclusions), and serious liver- (> 3 times the upper limit of reference value) or renal diseases (creatinine > 180 μmol/L) or hematological abnormalities (hemoglobin ≤ 7.0 mmol/L, hematocrit ≥ 0.50) (n = 27 exclusions). There were still 47 participants who met one of the other exclusion criteria and 25 participants who did not want to start intervention for other reasons. If participants did not match the study-profile due to one or more exclusion criteria, appropriate steps were undertaken to refer (if necessary) the participant to his general practitioner (n = 96). Major reasons for exclusion were testosterone level > cut-of-point, fasting glucose > 6.9 mmol/L or an elevated PSA level. Also, a fasting blood sample for a specific panel of laboratory assessments (including a spare DNA blood sample) was taken. Finally, 241 men proceeded to the randomization visit.

### Randomization visit

The randomization visit assessment was conducted when all lab results were known and always within 4 weeks from the information and screening visit. First, digital rectal examination and trans-rectal ultrasound of prostate were performed. If there was any suspicion regarding prostate pathology, appropriate steps were undertaken to refer the participant to its general practitioner or consulting urologist. Four men were excluded for this reason. If the rectal ultrasound did not show any signs of pathology the participant continued the following baseline measurements: medical history, family medical history, vital signs (blood pressure, pulse), physical examination, anthropometry measurements (height and body weight, waist- and hip circumference, upper leg-, arm- and calf circumference, sagittal abdominal diameter), International Prostate Symptom Score (IPSS) questionnaire, functional mobility measurements, bone mineral density measurements via DEXA scan, health related quality of life questionnaires and utilities instrument, measuring cognition (15-words test, Digit symbol Substitution test, Concept Shifting Task test, the Benton Judgment of Line Orientation test and the Shephard rotation task), full body DEXA scan (lean body mass, fat free and fat mass), Pulse Wave Velocity (PWV), abdominal ultrasound for fat distribution.

Finally 237 men were eligible for randomization. These subjects were randomly assigned to the intervention or the placebo group. A randomization list was computer-generated by Organon NV, Oss, The Netherlands. One box with active medication and one box with placebo medication were delivered at the UMC Utrecht Pharmacy with the randomization list. Pharmacy personnel labeled the jars for the participants and provided the study medication upon prescription of the trial physician. Randomization numbers were assigned to the subjects in orders of enrolment into the trial.

### Intervention

The intervention consisted of four capsules of 40 mg testosterone undecanoate (Andriol Testocaps^®^) provided by Organon NV, Oss, the Netherlands. The placebo was an identically looking and tasting capsule. The duration of the intervention was 26 weeks in which participants had to take the supplement on a daily basis. If for one reason unbinding should be necessary during the course of the trial, a backup hospital pharmacist was available who was informed about the trial but not involved.

### Control phone call (6 weeks)

All participants were called at 6 weeks after randomization. At 6 weeks participants were asked about: Medical history update, including co-medication and adverse events. Also, they filled in the IPSS questionnaire.

### Control visit (13 weeks)

During the intervention period of 26 weeks, subjects were asked to visit our clinic at 13 weeks after randomization (table 2). At 13 weeks the following measurement were done: Medical history update, including co-medication, adverse events, vital signs, (blood pressure, pulse), digital rectal examination, IPSS questionnaire and laboratory measurements (PSA, hematology, liver and renal functions, spare blood sampling for additional investigations).

**Table 2:** Baseline characteristics of participants

	Testosterone (n = 120)	Placebo (n = 117)
Age (yr)	67.3 ± 5.1	67.5 ± 5.0
Weight (Kg)	86.1 ± 13.3	84.4 ± 13.6
Body Mass Index (kg/m^2^)	27.54 ± 3.85	27.20 ± 3.90
Smokers	21 (17.5)	15 (12.8)
Alcohol users	99 (82.5)	85 (77.3)
Hypertension	75 (62.5)	69 (59)
Systolic Blood Pressure (mm/Hg)	156.2 ± 23.2	151.1 ± 22.6
Diastolic Blood Pressure (mm/Hg)	89.8 ± 12.0	86.8 ± 11.7
Pulse Pressure (mm/Hg)	66.4 ± 15.9	64.3 ± 15.2
Pulse Wave Velocity (m/s)	10.00 ± 2.51	9.53 ± 2.66
Total Testosterone (nmol/L)	10.93 ± 2.06	10.50 ± 1.89
Free Testosterone (nmol/L)	0.22 ± 0.05	0.21 ± 0.05
Bioavailable Testosterone (nmol/L)	5.23 ± 1.15	5.04 ± 1.20
SHBG (nmol/L)	33.17 ± 10.59	32.90 ± 10.38
Albumin (g/L)	43.94 ± 2.31	43.80 ± 2.38
Cholestrol (mmol/L)	5.61 ± 0.99	5.50 ± 0.97
HDL (mmol/L)	1.16 ± 0.28	1.16 ± 0.29
LDL (mmol/L)	3.92 ± 0.91	3.80 ± 0.87
Insuline (mIU/L)	10.14 ± 9.50	8.73 ± 5.41
C-reactive proteine (mg/L)	4.27 ± 6.56	4.09 ± 6.97
PSA (μg/L)	1.54 ± 1.1	1.63 ± 1.1
Creatinine (μmol/L)	93.35 ± 18.0	93.72 ± 15.2
ASAT (U/L)	22.98 ± 8.1	24.21 ± 12.2
ALAT (U/L)	26.36 ± 11.0	26.74 ± 13.6
AF (U/L)	71.57 ± 19.2	69.91 ± 17.9
GGT (U/L)	29.51 ± 15.751	30.18 ± 19.9
Hemoglobine (mmol/L)	9.18 ± 0.5	9.14 ± 0.6
Hematocrit (%)	0.45 ± 0.0	0.45 ± 0.0
Prostate Volume (ultra sound) (cc)	28.20 ± 12.4	27.6 ± 9.8
IPSS	6.38 ± 5	6.50 ± 4.8

Values are means or numbers with S.D. or percentage in parentheses.

### Final visit (26 weeks)

The final visit took place after 26 weeks of intervention. At the final visit, all tests carried out at base-line were repeated following the same procedures.

### End point measurements

An overview of visits and measurements is shown in Tables 2 and 3, respectively. Endpoints were assessed at baseline and after 26 weeks. All the assessments took place at the baseline randomization visit were repeated at 26 weeks.

**Table 3:** Overview of the visits and measurements

Assessment	Information visit and inclusion criteria	Screening and randomization visit	Phone call 6 weeks	Visit 13 weeks	Final visit 26 weeks
	V1	V2		V3	V4
Check in & exclusion criteria	X	X			
Informed consent	X				
Medical history (/update)	X	X	X	X	X
Physical examination		X			X
Vital signs		X		X	X
Anthropometry		X			X
Blood sample	X			X	X
Glucose	X				X
DNA blood sample	X				
Testosterone	X				X
PSA	X			X	X
Liver functions	X			X	X
Renal functions	X			X	X
Hematology	X			X	X
Spare blood	X			X	X
Rectal Ultrasound		X			X
Rectal Toucher		X		X	X
DEXA full body		X			X
DEXA bone mineral density		X			X
Ultrasound abdominal fat mass		X			X
Pulse wave velocity (PWV)		X			X
Functional mobility		X			X
Cognition tests		X			X
Quality of Life (QoL)		X			X
ADAM/AMS questionnaire	X				X
Sexual functioning questionnaire		X			X
IPSS questionnaire		X	X	X	X
Randomization		X			

### Functional mobility measurement

We assessed the functional mobility by the use of timed "Get Up and Go" test and a questionnaire for the ability to perform activities of daily life the Stanford Health Assessment Questionnaire (HAQ)[25,26]. Furthermore, skeletal muscle strength was assessed measuring handgrip strength and isometric knee strength [27,28].

During the timed "Get Up and Go" test, the time taken by an individual to rise from a standard chair, walk three meters, turn around, return and sit down again was measured. The subject was requested to sit with his back against the chair and arms resting on the chair and performs the test three times. The fastest time was recorded in seconds.

The Stanford Health Assessment Questionnaire (HAQ) has been widely used to measure functional status and includes 24 questions grouped into 8 categories of 2, 3 or 4 ADL's. The categories were dressing, arising, eating, walking, hygiene, reaching, gripping, and others. Participants responded to these questions by checking the level of difficulty from 0 (without any difficulty) till 3 (unable to do). If participants needed help from another person or assistive devices for each of the ADL's the score raises automatically to 2 (with much difficulty).

Handgrip strength was measured with the JAMAR® dynamometer. The size of the grip was set so that the participant felt comfortable. The participant was in standing position his shoulder was adducted and neutrally rotated; the arm was vertical and the wrist in a neutral position. The participant squeezed the grip with maximal strength, alternating the left and right hand. The unit was automatically recorded the highest force exerted. Each test was repeated at least 5 times until no further improvements were seen. The best measure, recorded in kilograms, was used for analysis.

Isometric knee extensor strength was measured with a hand-held dynamometer. The participant was in a seated position at a mat-table with the hip flexed to 90 degrees, the knee stretched to 180 degrees and the legs dependent. The dynamometer was applied perpendicularly to each lower extremity just proximal to the malleoli. Participants were instructed to take a second or two to come to come to maximum effort and to then push as hard as possible during another three seconds, while the investigator was giving counterforce. Each test was repeated five times, and if the examiner was not confident that a maximal effort was reached one more effort was made. The best measure, recorded in Newton, was used for analysis.

### Quality of life and well-being measurement

Quality of Life and well being was measured by the Short Form-36 Health Survey as a generic QoL questionnaire (SF-36) and the Herschbach-questionnaire as a hormone specific questionnaire. The SF-36 is a questionnaire consisting of questions regarding general health, ability to perform physical activity and work, emotional problems and assessment of his own health [29]. The Herschbach questionnaire is a questionnaire translated from the questionnaire "Fragen zur Lebenszufriedenheit" (FLZ) according to the method described by Henrich and Herschbach. The questionnaire is divided in a "general" and a "health" section, each including eight items. All items have been evaluated on a 5-point scale according to their individual importance (I) and degree of satisfaction (S). As a measure of evaluation, a combination of importance and satisfaction (I-1)*(S*2–5) will be used. In addition the sum of the combination values will be calculated for each section [30].

### Sexual behavior and erectile dysfunction measurement

The 'Eleven questions about sexual functioning (ESF) questionnaire, developed by the National Institute for Social Sexual Research (Rutgers Nisso Group, Utrecht, The Netherlands), has been used to assess sexual well being. The questionnaire has 11 questions measuring sexual drive (two questions); erectile function (three) and ejaculatory function (two), as well as assessing problems with sex drive, erections, or ejaculation (three); and overall satisfaction with sex life (one). Each question is scored on a scale of 0–4, with higher scores indicating better functioning.

The Androgen Deficiency in Ageing Males (ADAM) and the Ageing Males' Symptoms rating scale (AMS) questionnaires have been administered as well. The ADAM questionnaire contains 10 questions regarding the age-related decline in androgens. All questions should be answered yes or no. A positive questionnaire result, indicating an androgen deficient state, is defined as a 'yes' answer to question 1 or 7 or any 3 other questions [31]. AMS-questionnaire (Ageing Males' Symptoms rating scale) is a 17-question questionnaire investigating age related health complaints divided in three dimensions (psychological, somatovegetative and sexual) of each 5 questions. Each question can be scored from 1 (no symptoms) to 5 (very severe symptoms), so a dimension can score from 5 to 25 points. Within each dimension, cumulative scores indicate the severity of the complaints on each territory; also the cumulative of all dimensions indicate an overall view of Ageing Males' Symptoms. Classification range spreads from no impairment at all to severe impairment [32].

### Body composition measurement

Body Composition was assessed by anthropometry (body mass index (BMI), waist and hip girth, upper arm-, upper leg and calf circumference and sagittal abdominal diameter), full body DEXA scan (lean body mass, fat free- and fat mass) and ultrasound of the abdominal fat mass.

BMI was calculated as the weight in kilograms divided by the square of the height in meters, after taking of coat, sweaters and shoes. All circumference measurements were done with a standard household centimeter. Waist circumference was measured at the level of midway the distance between the lower rib and iliac crest, after normal expiration without pressure of the centimeter at the skin. The hip circumference was measured at the level of the greater trochanter. The upper arm circumference was measured at the non-dominant arm at the level of midway between the tip of the acromnion and the olecranon. The thigh circumference was measured just below the gluteal fold of the left leg. Calf circumference was measured at the level of the largest circumference of the left calf. The Sagittal abdominal diameter (SAD) was measured using a Holtain-Kahn (abdominal calliper (Holtain ltd., Crosswell, UK) which allows a direct reading of the distance between the subjects back and the front of the subjects' abdomen. With subject in supine position a mark was made halfway between the left and right iliac crest. The lower arm of the caliper was inserted underneath the subjects back and the upper arm was adjusted until touching the abdominal wall at the level of the mid-abdominal mark. The measurement was taken with a resting and at the end of a normal expiration. The distance between the subjects back and abdominal wall was measured on a centimeter scale and round off to the nearest 0.1 cm. Since with the abdominal subcutaneous fat tends to slip along the flanks, when the subject is in supine position, the SAD is an indirect measurement of the amount of visceral fat mass.

Total body composition was measured with dual energy X-ray absorptiometry (DEXA) using a Lunar prodigy^® ^DEXA instrument. Scanning was performed according to the instructions of the manufacturer. After placement of the subject on the table, there was scanning of the whole subject from dorsal to ventral. Both legs and feet were endorotated and fixed to on another. Calculations were made regarding fat-mass, fat-free mass and lean body mass [33].

Abdominal ultrasonography was performed in all abdominal obese subjects with a Ultramark 9^®^. The distances between the posterior edge of the abdominal muscles and the lumbar spine or psoas muscles were measured using electronical callipers. For all images the transducer was placed on a straight line drawn between the left and right mid-point of lower rib and iliac crest. The middle was marked 10 cm from the left and right side. Distances were measured from three different angles: medial, left and right for intra-abdominal fat mass and medial for subcutaneous fat mass. Measurements were made at the end of quiet expiration, applying minimal pressure without displacement of intra-abdominal contents as observed by ultrasound image [34].

### Cognitive function measurement

Cognitive function was measured as follows. Verbal memory was tested with the Dutch version of the Rey Auditory Verbal Learning Task. This is a test for long-term memory retention. Fifteen words were read to the subject, who was required to report as many words as he could remember immediately after presentation. After a delay of 15 min (in which another test, the Benton Judgment of Line Orientation, was administered), the subject was asked to recall as many words as possible from memory [35].

Mental processing speed was tested with the "Digit Symbol Substitution test". This is a sub-test from the Wechsler Adult Intelligence Scale (WAIS) that covers general knowledge. It measures cognitive and perceptual-motor processing speed. The subject was given a code that pairs symbols with digits. The test consists of matching as many series of digits to their corresponding symbols as possible in 90 sec [36].

The trail making test was used to test planning of movement, vasomotor tracking, and processing speed. In this test, pseudo-randomly placed circles with numbers (Trail Making A1), with letters (Trail Making A2), and with both numbers and letters (Trail Making B) have to be connected with a line as fast as possible in a fixed order. In the event of error, the subjects were immediately informed and asked to restart from the point of error: this was done with the timer left running. The time taken to complete the trail without error was recorded [37]. The "Benton Judgment of Line Orientation test" was used to measure visual-spatial skills. This test measures basic perceptual processes contributing to extra-personal spatial perception. The test requires the subject to identify which 2 of 11 lines presented in a semicircular array have the same orientation in two-dimensional space as two-target lines [38].

The visospatial performance was assessed by the Vandenberg and Kuse adaptation of Shepard and Metzler's three-dimensional mental rotations test [39]. This is the cognitive task that has been most consistently associated with testosterone levels. The test was consisted of 20 items in which the subject was presented with a three-dimensional geometric target line drawing and four test drawings, and was required to indicate which two of four test drawings depict the target drawing in rotated positions. Two parallel test versions were made by taking the odd and even items on time 1 (baseline) and time 2 (after intervention) respectively (10 items for each test). These parallel versions have been shown to correlate strongly with each other and to have a high reliability. Subjects were instructed to "work as quickly as possible, but do not sacrifice accuracy for speed". They were allowed 10 minutes to complete the test.

### Aortic stiffness and cardiovascular risk factors measurements

Total cholesterol, HDL cholesterol and triglyceride were measured by a timed endpoint method (Synchron LX^®^, Beckman Coulter, Fullteron, California, USA) [40]. LDL was calculated with the Friedewald equation [41]. Insulin was measured by a solid-phase two site chemiliminesent immunometric assay (IMMULITE 2000, Diagnostic Products Corporation, Los Angeles, California, USA). Serum levels of highly sensitive CRP were measured using a near-infrared particle immunoassay of the Synchron LX System (Synchron LX^®^, Beckman Coulter, Fullteron, California, USA).

Systolic and diastolic blood pressures and pulse were measured in duplicate at the dominant arm with the subjects in sitting position after 5 minutes of rest with an automated and calibrated oscillometric device (Omron Healthcare Europe, Hoofddorp, The Netherlands). Subsequently, the mean systolic and diastolic blood pressures and mean pulse rate were calculated.

Aortic stiffness was determined by means of pulse wave velocity. The Sphygmocor® system was used to non-invasively measure stiffness of the aorta (Pulse wave velocity system, PWV medical, Sydney, Australia) [42]. After 5 to 10 minutes rest of the subject in supine position, aortic PWV was measured by sequentially recordings of arterial pressure waveform at the carotid artery and the femoral artery using a hand-held micromanometer-tipped probe on the skin at the site of maximum arterial pulsation. Gating the recordings at those two sites to the electrocardiogram (ECG) allowed PWV to be measured. Recordings were taken when a reproducible signal was obtained with high amplitude excursion, i.e. usually 10 consecutive beats to cover complete respiratory cycle. The system software, using the R wave of a simultaneously recorded ECG as a reference frame, was calculated the wave transit time. A distance from the carotid-sampling site to the suprasternal notch and suprasternal notch to the femoral artery was measured using a compass [43].

### Bone mineral density measurement

Bone mineral density (BMD) was measured with dual energy X-ray absorptiometry (DEXA) using a Lunar prodigy® DEXA instrument. Scanning was performed according to the instructions of the manufacturer. BMD was measured of lumbar vertebrae (L1–L4 individually and together) and proximal femur (femoral neck, trochanter, inter-trochanter, Ward's triangle and total hip, left-or right if left not available). A T-score≤-2.5 denotes osteoporosis, a T-score between -1 and -2.5 denotes osteopenia [44]. The DEXA scan was also used to measure total and trunk lean body mass (see total body DEXA-scan). Quality assurance, including calibration was performed routinely every morning for DEXA (if that day a measurement is planned), using the standard provided by the manufacturer [45].

### Prostatic measurements

Effects on the treatments on the prostate were examined by digital rectal examination, transrectal ultrasound of the prostate and by monitoring serum prostate-specific antigen (PSA) levels and by IPSS. The IPSS, developed by the American Urological Association (AUA), contains seven items that measure frequency and severity of urological symptoms, together with an additional item measuring the overall impact of these symptoms on quality of life. Each of the seven symptom items has a response scale with six choices, scored from 0 (absence of the symptom) to 5 (symptom always present). Symptoms are considered mild for scores between 0 and 7, moderate for scores between 8 and 19, and severe for scores between 20 and 35 [46].

Digital rectal examination was performed at baseline, 13 weeks and at the end of treatment (26 weeks). Biplanal transrectal ultrasonography of the prostate was performed at baseline and at the end of treatment (26 weeks) with a 7-MHZ transrectal probe (Bruel and Kjaer Model 2110 Falcon). If rectal ultrasound was abnormal, patients were excluded and referred for further evaluation. Serum prostate specific antigen (PSA) levels were measured by an immunnometric assay (IMMULITE^® ^2000 PSA, Diagnostic Products Corporation, Los Angeles, California, USA) at baseline, week 13 and at the end of the study. An increase of > 1.4 μg/L between measurements at any time was cause for concern. Abnormal values required repeat testing; if values remained high, co-morbid illness was ruled out. This was reason to exclude a patient and send to his general practitioner.

### Laboratory measurements

Fasting blood samples were obtained between 8.00 and 11.00 AM to minimize diurnal variation. The level of total testosterone and sex hormone binding globulin (SHBG) were measured with a solid-phase, competitive, chemiluminescent enzyme immunoassay (IMMULITE ^® ^2000, Diagnostic Products Corporation, Los Angeles, California, USA) at baseline and at the end of the study. The intra-assay coefficient of variation of this assay was 7.2% and the inter-assay coefficient of variation was 8.2 % for testosterone and 2.5% and 5.2 % for SHBG, respectively. Hematology (hemoglobin and hematocrit) and routine biochemistry (liver functions and creatinine) were measured by standard autoanalyzer methodologies (Synchron LX^®^, Beckman Coulter, Fullteron, California, USA) at baseline, after 13 weeks, and at the end of the study. During the study, hemoglobin levels of ≤ 7 mmol/L, hematocrit levels ≥ 0.50, liver function values ≥ three times normal upper normal reference level (ASAT: 15–45 U/L; ALAT: 10–50 U/L; AF: 40–130 U/L ; γ-GT: 15–70 U/L), or creatinine levels of ≥ 180 μmol/l led to an extra blood check after a week. If the values were still too high study participation was discontinued. All laboratory measurements were done at the SHO laboratory, Velp, The Netherlands.

### Adverse events

An adverse event (AE) was defined as any untoward medical occurrence in a patient or clinical investigation subject administered a pharmaceutical product and which did not necessarily have a causal relationship with this treatment. An AE could therefore be any unfavorable and unintended sign (including an abnormal laboratory finding), symptom, or disease temporarily associated with the use of a medicinal report, whether or not considered related to the medicinal product. Whether or not an abnormal laboratory/vital sign were entered on the AE form depends on whether or not the finding was clinically relevant in the opinion of the investigator. Information regarding AEs was obtained by questioning or examining the subject. At each visit during the treatment period, all new complaints and symptoms (i.e. those not existing before the treatment period) were recorded (and coded) on the AE Form. Pre-existing complaints or symptoms that increased in intensity or frequency during the treatment period were entered on the AE Form also. All AEs were characterized in terms of their start and stop dates, maximum intensity, action taken on trial medication, relationship to trial medication, and subject outcome. If a subject discontinued the trial because of an AE, this was noted on the AE Form. Serious adverse events (SAE) forms were supplied by Organon. If the AE meets the definition of an SAE, the procedure for reporting SAEs was followed. A serious adverse event (SAE) was defined as any untoward medical occurrence that at any dose: resulted in death, was life-threatening, required in-patient hospitalization or prolongation of existing hospitalization, resulted in persistent or significant disability or incapacity, noted the term life threatening refers to an event in which the patient was at risk of death at the time of the event; it did not refer to an event, which hypothetically might have caused death if it had been more severe. Medical and scientific judgment was exercised in deciding whether expedited reporting was appropriate in other situations, such as important medical events that might not be immediately life-threatening or result in death or hospitalization, but may jeopardized the patient or might required intervention to prevent one of the other outcomes listed in the definition above. These were considered serious. All SAEs were reported to the METC and to Organon NV, Oss, the Netherlands. Every attempt was made to obtain any relevant laboratory or hospital reports that pertain to the SAE.

### Compliance

Compliance was monitored by spare capsule counting at each study visit. After finalization of the study serum testosterone concentrations were assessed in the final visit blood samples as an extra check on compliance.

### Power calculation

The pre-specified number of subjects was 240 in total, 120 in each intervention arm. This number was based on conventional assumptions of α = 0.05 and β = 0.20, withdrawal from intervention of 15% and an improvement of 25% on MHAQ and of 18% on the 15 Words test. These improvements were realistic, since they have been previously reported in short-term small studies.

### Data analysis

The primary analysis will be done by linear regression analysis with change in outcome parameter between final visit and baseline visit as the dependent and treatment group as the independent variable. All analyses will be based on an intention-to-treat approach (i.e., the intention-to-treat group will consist of all subjects, including those who withdrew from blinded medication, who received at least one dose of study drug and who had at least one post-baseline assessment of the outcome variable). In addition to an intention-to-treat analysis, a per-protocol analysis will be performed. The per-protocol group will consist of all subjects from the intention-to-treat group who did not have any major protocol violations. Furthermore, subgroup analysis will be performed for the following predefined subgroups according to baseline measurements: waist girth (< 100 cm versus ≥ 100 cm); testosterone level (< 12 versus ≥ 12 nmol/L), age (< median versus ≥ median), and baseline level of outcome under study (< median versus ≥ median). Differences between final visit and baseline for continuous measures were expressed as means and 95% confidence intervals; unpaired t-tests were used for testing. Level of significance was set at P < 0.05. All analyses are performed with SPSS, statistical software package, version 11.

## Competing interests

The author(s) declare that they have no competing interests.

## Authors' contributions

YTvdS, HJJV and DEG formed the original study team that developed the research question, wrote the study protocol obtained local ethics approval, obtained grant funding and implemented this study. HRNP, MHEV and MSH participated in its design and coordination. HRNP drafted the manuscript. MHEV, HJJV, MSH, DEG, and YTvdS helped to draft the manuscript. All authors read and approved the final manuscript.
